# Supplementary material for: From small-scale forest structure to Amazon-wide carbon estimates
Source: Nat Commun. 2019 Nov 8;10:5088. doi: 10.1038/s41467-019-13063-y (PMC6841659; doi:10.1038/s41467-019-13063-y)
Supplement: Supplementary file 1 — Supplementary Information [file 41467_2019_13063_MOESM1_ESM.pdf]

Supplementary Information

From small-scale forest structure to Amazon-wide carbon estimates.

Rödig et al.

Supplementary Figures

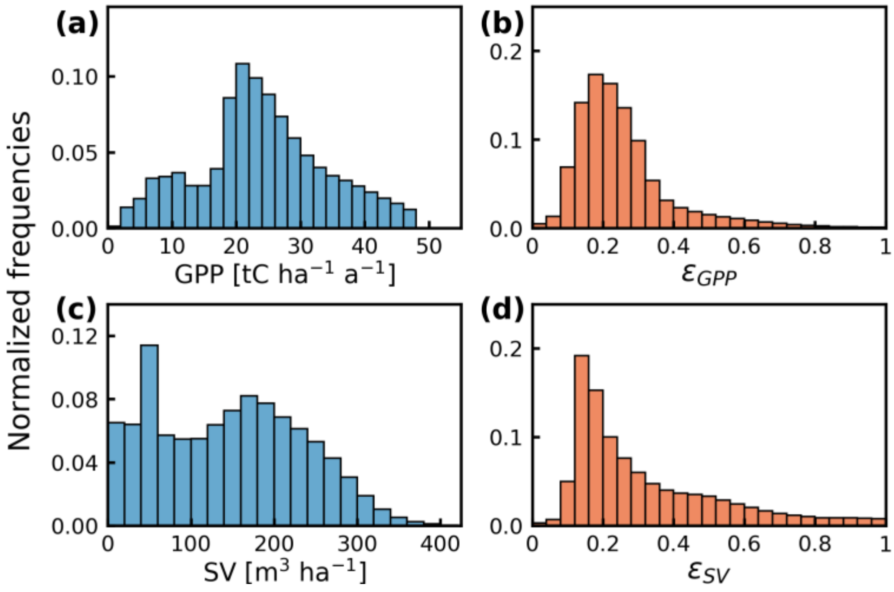

Supplementary Fig. 1: Frequency distributions of forest attributes and uncertainties. Normalized frequency distributions of (a) gross primary productivity (GPP) and (c) stem volume (SV) taking 771,521 full lidar profiles in the Amazon as a proxy for forest attributes. Normalized frequency distributions of the corresponding uncertainty indices (b) ε_{GPP} for productivity and (d) ε_{SV} for stem volume.

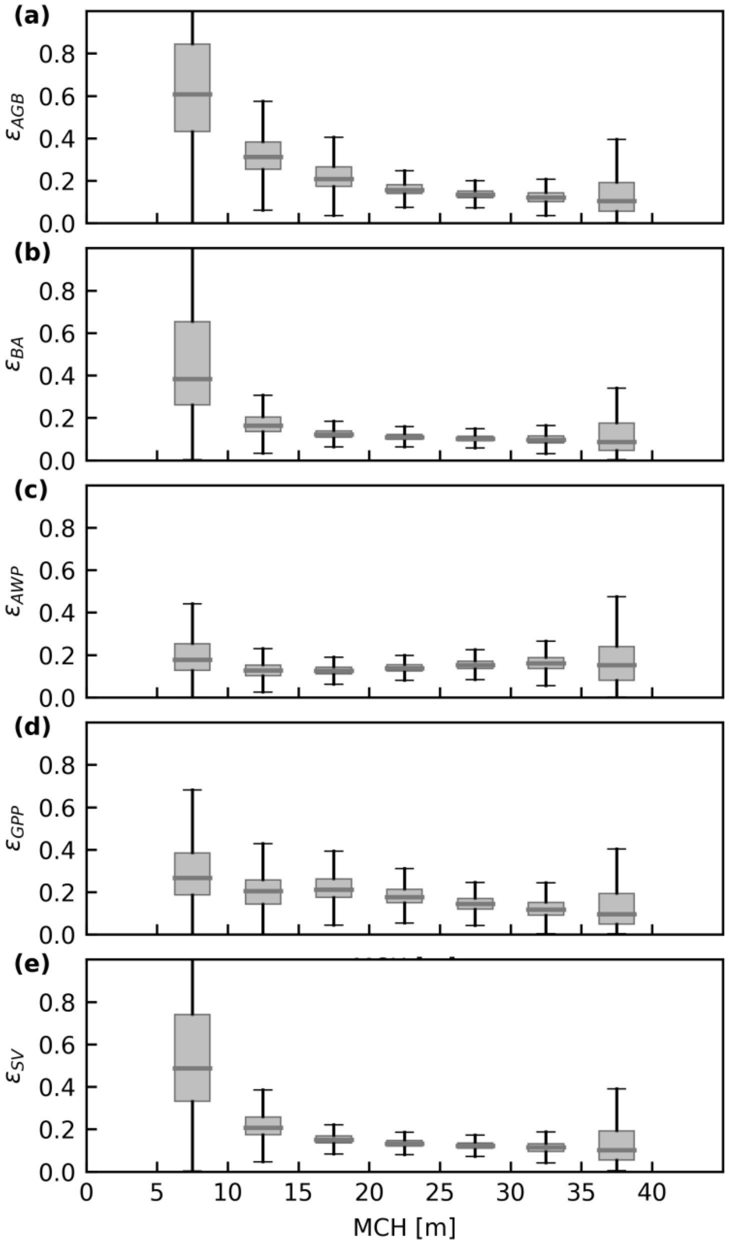

Supplementary Fig. 2: Uncertainty indices ε for different forest attributes. Shown are ε of (a) aboveground biomass (AGB), (b) basal area (BA), (c) aboveground woody productivity (AWP), (d) gross primary productivity (GPP), and (e) stem volume (SV) versus mean canopy height (MCH).

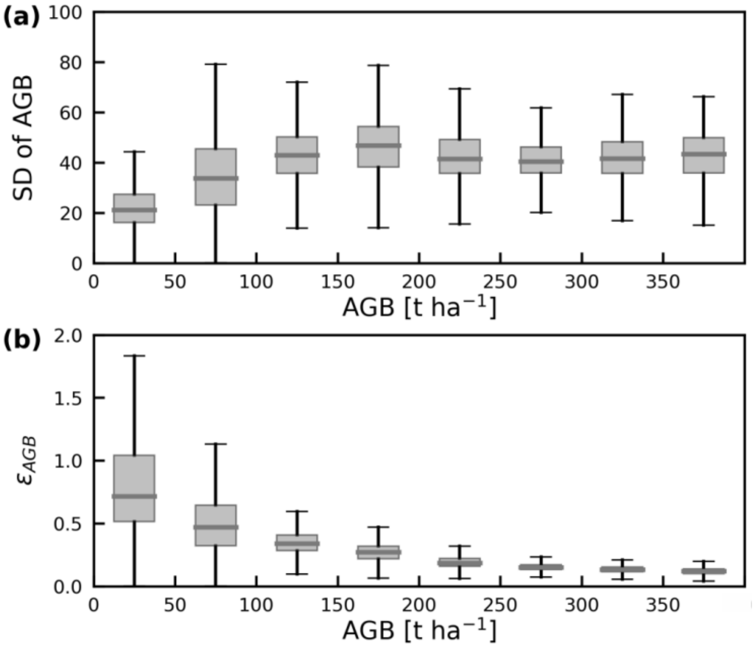

Supplementary Fig. 3: Uncertainties of aboveground biomass (AGB) in different AGB classes. (a) Mean standard deviation (SD) of probability distributions of AGB versus AGB classes. (b) Uncertainty index of AGB ε_{AGB} (mean coefficient of variation of probability distributions) versus AGB classes. The grey line marks the median, whiskers range across the first and third quartiles.

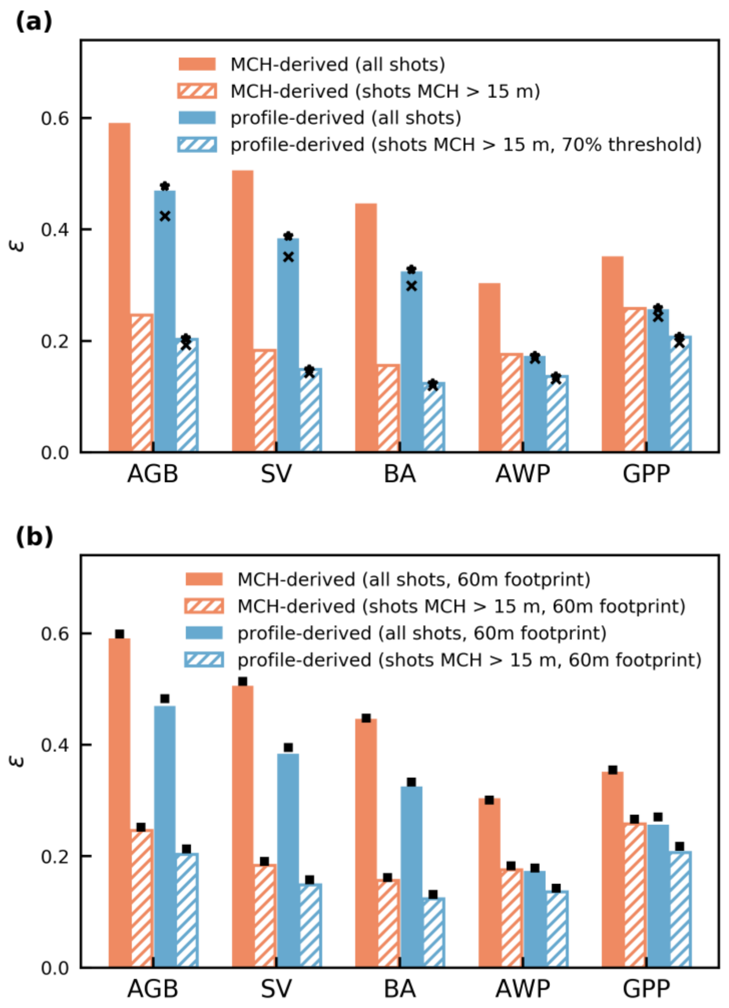

Supplementary Fig. 4: Mean uncertainty ε for forest attributes using different estimation approaches. Estimations were based on either taking mean canopy height as a proxy for forest attributes (MCH-derived, orange) or taking the entire lidar profile as a proxy (profile-derived, blue). Shown are ε for above-ground biomass (AGB), basal area (BA), aboveground woody productivity (AWP), gross primary productivity (GPP), and stem volume (SV) for all lidar profiles and for lidar profiles with a mean canopy height (MCH) < 15 m. (a) Boxes represent results for a threshold of 70%. The stars indicate the model uncertainty for a threshold of 60% and the crosses for a threshold of 80%. With a 60% threshold, ε is barely higher than for a 70% threshold. That means that most of the lidar shots match 50 shots with a relative overlap of greater than 70%.With an 80% threshold, ε slightly decreases. That means that some lidar shots match less than 50 shots with a relative overlap of greater than 80%, reducing the

uncertainty index ε by 1-4%. When only taking shots with a MCH >15 m into account, the results differ by less than 0.5%. Our analyses show that the uncertainty index ε is mainly independent of the relative overlap (Supplementary Fig. 8). (b) Boxes represent results for a threshold of 70% with a 65-m footprint (as in Fig. 5). The squares indicate the uncertainty index ε for a 60-m footprint.

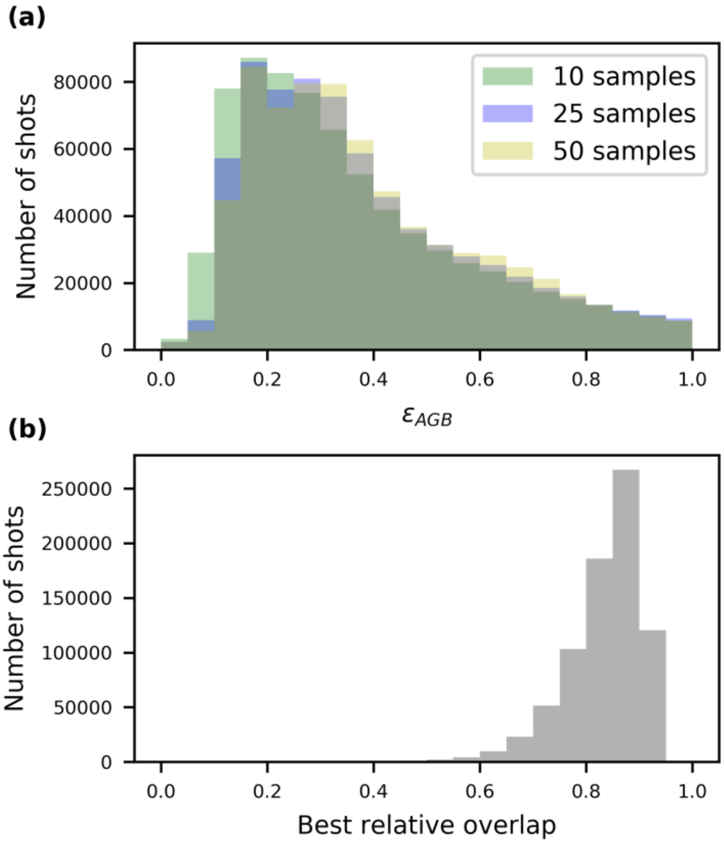

Supplementary Fig. 5: (a) Frequency distribution of the uncertainty index of aboveground biomass (AGB) ε_{AGB} for the 10, 25, and 50 best matches with lidar profiles. (b) Frequency distribution of mean best relative overlaps.

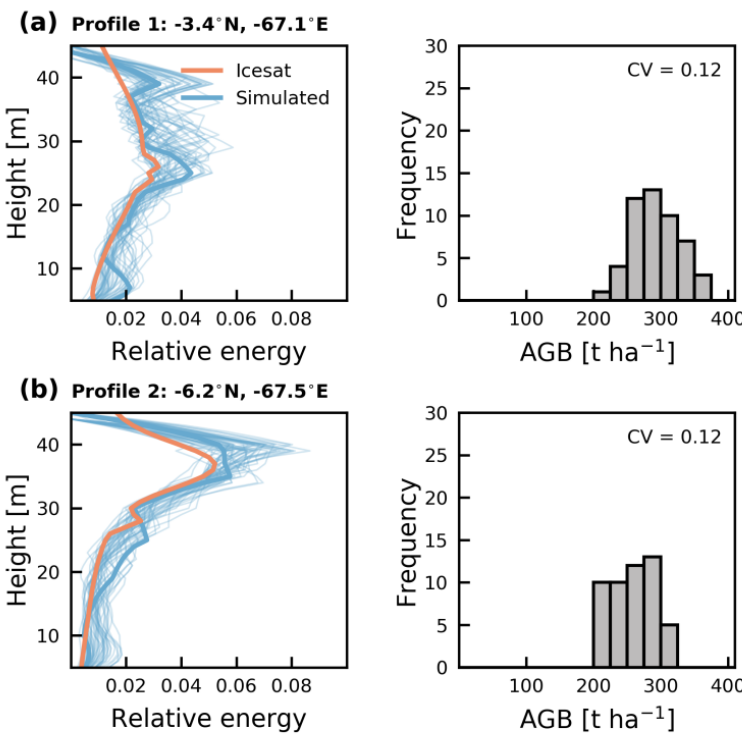

Supplementary Fig. 6: Two exemplary locations where GLAS lidar profiles were compared with simulated forests: at (a) -3.4°N, -67.1°E, and (b) -6.2°N, -67.5°E. (left) Reconstructed GLAS lidar profile (red) and best matching simulated profiles (blue for best relative overlap, light blue for the other 49 simulated profiles with highest overlap). The mean relative overlap of (a) is 72% and (b) 73%. (right) Above-ground biomass (AGB) distribution for 50 simulated profiles.

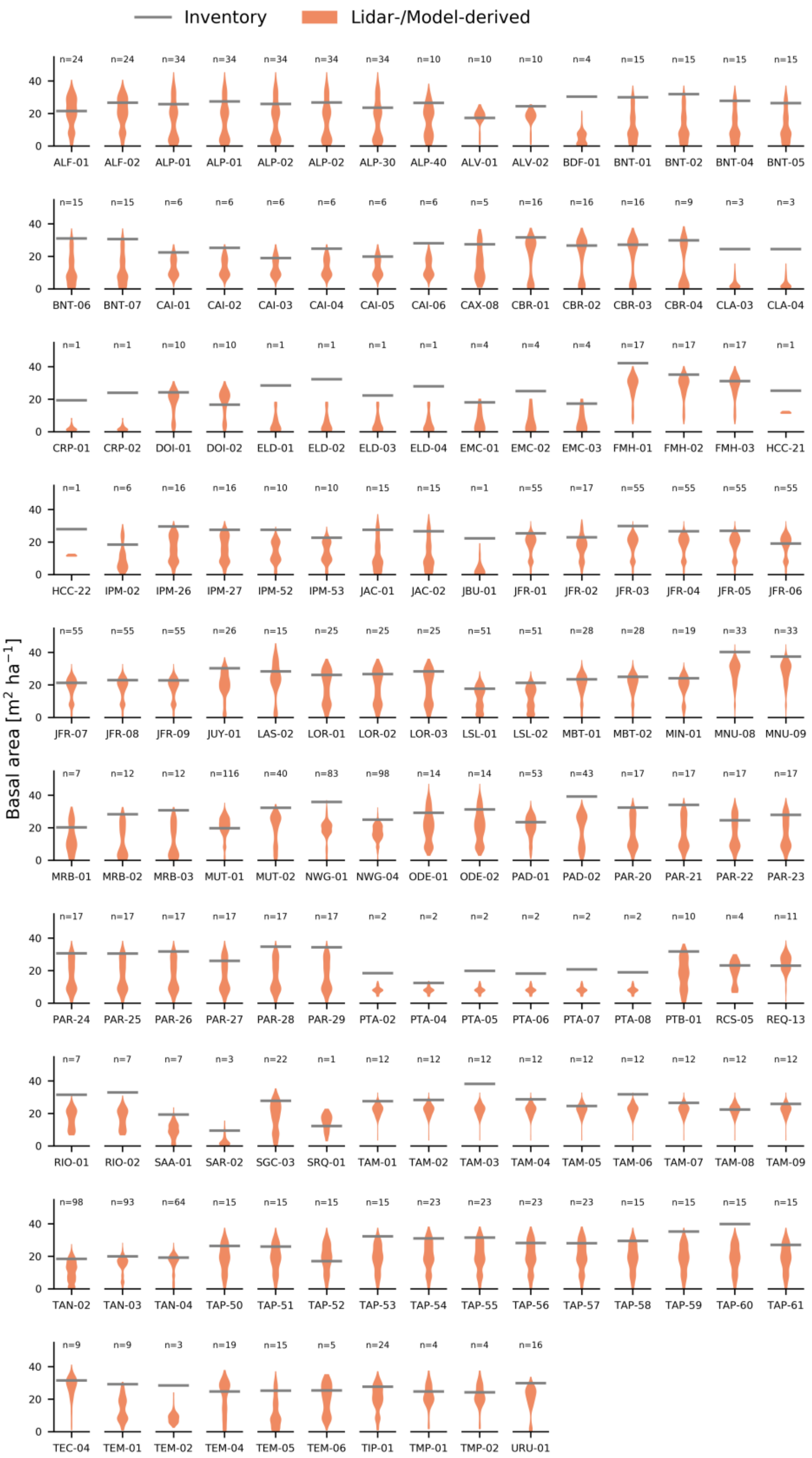

Supplementary Fig. 7: Comparison of observed basal area at 140× 1-ha inventory plots[1,2] against simulated values (only trees with diameter > 10 cm) derived from the nearest lidar shots (n indicate the number of shots) within a 3 km radius around inventory (uncertainty of the coordinates of the inventory sites[3]).

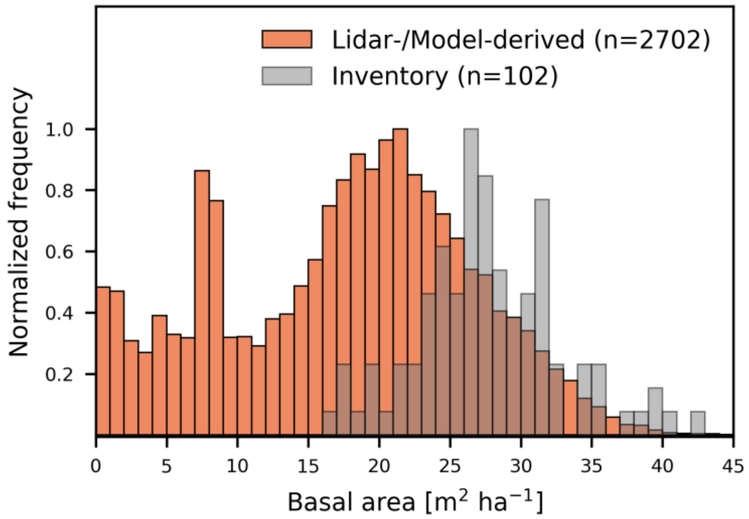

Supplementary Fig. 8: Normalized (by maximum) frequency distribution of (grey) observed basal area (tree diameter > 10 cm) for 102 inventory plots that were surrounded by at least 10 lidar shots within a 3km radius, and (red) lidar-/model derived basal area (tree diameter > 10 cm) for 2702 lidar shots that fall into those radii. Due to the 'bias towards majestic forest stands' for field inventories[4], values derived from inventory data tends to be higher than the values derived from random lidar shots, because ICESat also captures disturbed forest stands. Overall however, the lidar shots cover the entire range that is covered by inventories.

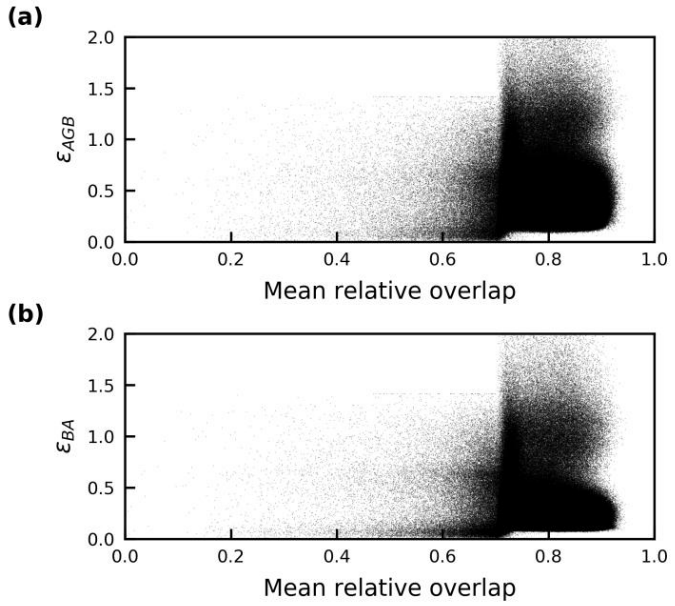

Supplementary Fig. 9: Uncertainty index ε plotted against relative overlap. (a) For aboveground biomass (AGB) ε_{AGB}, and (b) for basal area (BA) ε_{BA}.

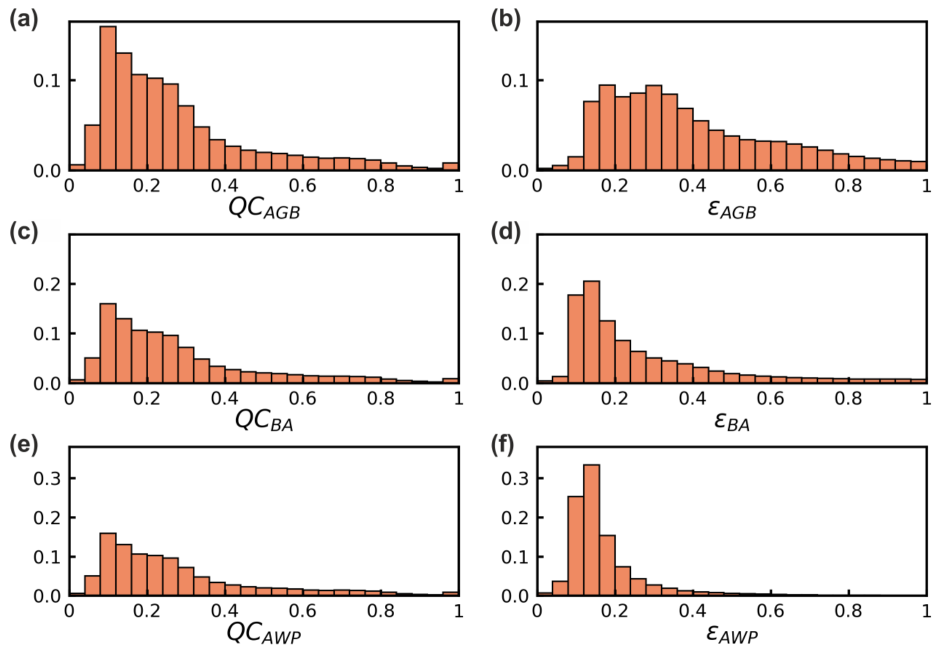

Supplementary Fig. 10: Frequency distributions of uncertainty of different forest attributes. Shown are results using two different uncertainty definitions: (left) the quartile coefficient of dispersion (QC) and (right) the coefficient of variation (uncertainty index ε) for (a, b) above-ground biomass (AGB), (c, d) basal area (BA), and (e, f) aboveground wood productivity (AWP) for the Amazon based on 771,521 full lidar profiles.

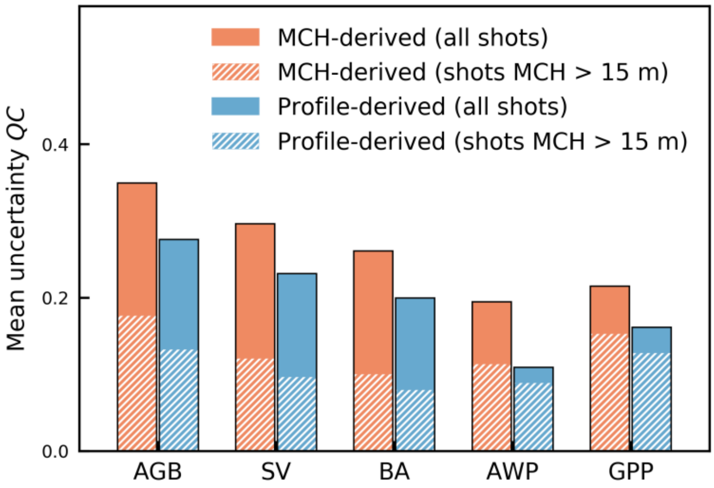

Supplementary Fig. 11: Mean uncertainties as the quartile coefficient of dispersion (QC) for different forest attributes. Shown are QC for aboveground biomass (AGB), stem volume (SV), basal area (BA), aboveground wood productivity (AWP), and gross primary productivity (GPP) taking mean canopy height (MCH) as a proxy for forest attributes (MCH-derived) vs. the entire lidar profile (profile-derived). Hatched bars show the mean uncertainty index for lidar profiles with a MCH > 15 m.

Supplementary References

1. Lopez-Gonzalez, G. *et al.* Amazon forest biomass measured in inventory plots. Plot Data from "Markedly divergent estimates of Amazon forest carbon density from ground plots and satellites. *www.forestplots.net* (2014). doi:10.5521/FORESTPLOTS.NET/2014_1

2. Mitchard, E. T. a. *et al.* Markedly divergent estimates of Amazon forest carbon density from ground plots and satellites. *Glob. Ecol. Biogeogr.* **23,** 935–946 (2014).

3. Saatchi, S. *et al.* Seeing the forest beyond the trees. *Glob. Ecol. Biogeogr.* **24,** 606–610 (2015).

4. Malhi, Y. *et al.* An international network to monitor the structure, composition and dynamics of Amazonian forests (RAINFOR). *J. Veg. Sci.* **13,** 439 (2002).
