# Supplementary material for: From small-scale forest structure to Amazon-wide carbon estimates
Source: Nat Commun. 2019 Nov 8;10:5088. doi: 10.1038/s41467-019-13063-y (PMC6841659; doi:10.1038/s41467-019-13063-y)
Supplement: Supplementary file 3 — Reporting Summary [file 41467_2019_13063_MOESM3_ESM.pdf]

nature research

Corresponding author(s): *Double-blind peer review submissions: write DBPR and your manuscript number here instead of author names.*

Last updated by author(s): *YYYY-MM-DD*

Reporting Summary

Nature Research wishes to improve the reproducibility of the work that we publish. This form provides structure for consistency and transparency in reporting. For further information on Nature Research policies, see Authors & Referees and the Editorial Policy Checklist.

Statistics

For all statistical analyses, confirm that the following items are present in the figure legend, table legend, main text, or Methods section.

n/a	Confirmed	
☐	☒	The exact sample size (*n*) for each experimental group/condition, given as a discrete number and unit of measurement
☒	☐	A statement on whether measurements were taken from distinct samples or whether the same sample was measured repeatedly
☒	☐	The statistical test(s) used AND whether they are one- or two-sided<br>*Only common tests should be described solely by name; describe more complex techniques in the Methods section.*
☒	☐	A description of all covariates tested
☒	☐	A description of any assumptions or corrections, such as tests of normality and adjustment for multiple comparisons
☐	☒	A full description of the statistical parameters including central tendency (e.g. means) or other basic estimates (e.g. regression coefficient) AND variation (e.g. standard deviation) or associated estimates of uncertainty (e.g. confidence intervals)
☒	☐	For null hypothesis testing, the test statistic (e.g. *F*, *t*, *r*) with confidence intervals, effect sizes, degrees of freedom and *P* value noted<br>*Give P values as exact values whenever suitable.*
☒	☐	For Bayesian analysis, information on the choice of priors and Markov chain Monte Carlo settings
☒	☐	For hierarchical and complex designs, identification of the appropriate level for tests and full reporting of outcomes
☒	☐	Estimates of effect sizes (e.g. Cohen's *d*, Pearson's *r*), indicating how they were calculated

Our web collection on statistics for biologists contains articles on many of the points above.

Software and code

Policy information about availability of computer code

Data collection	Remota sensing Lidar data were taken from Tang et al., 2017, PNAS.
Data analysis	Data were pre-, postprocessed and plotted with Python 2.7. Forest simulation data were simulated with the Amazon-wide version of FORMIND (Rödig et al., 2017). The FORMIND model is freely available on www.formind.org.

For manuscripts utilizing custom algorithms or software that are central to the research but not yet described in published literature, software must be made available to editors/reviewers. We strongly encourage code deposition in a community repository (e.g. GitHub). See the Nature Research guidelines for submitting code & software for further information.

Data

Policy information about availability of data

All manuscripts must include a data availability statement. This statement should provide the following information, where applicable:

- Accession codes, unique identifiers, or web links for publicly available datasets
- A list of figures that have associated raw data
- A description of any restrictions on data availability

Source data underlying Fig. 2, 3, 4 and Supplementary Figures are provided as a Source Data file.

Field-specific reporting

Please select the one below that is the best fit for your research. If you are not sure, read the appropriate sections before making your selection.

☐ Life sciences ☐ Behavioural & social sciences ☒ Ecological, evolutionary & environmental sciences

Ecological, evolutionary & environmental sciences study design

All studies must disclose on these points even when the disclosure is negative.

Study description	The study is based on simulations of an Amazon-wide forest model. These simulations were matched with 771,521 Lidar measurements taken onbord Icesat in order to identify the states of the forests. Combining the forest simulations with the Lidar data helps to identify several forest attributes (biomass, basal area, productivity, etc.).
Research sample	We used all available Lidar shots of Tang et al., 2017, PNAS within the Amazon region with a mean canopy height above 5m (771,521 shots in total).
Sampling strategy	No sampling strategy was needed.
Data collection	Forest simulation data were derived from the forest model FORMIND (Rödig et al. 2017). Lidar data were taken from Tang et al., 2017, PNAS.
Timing and spatial scale	Simulation results were analyzed at the spatial scale of the Lidar shots (~65m footprints). Lidar data were taken onboard Icesat in 2003-2006.
Data exclusions	We excluded all Lidar shots with a mean canopy height below 5m.
Reproducibility	Our study is not based on field experiments, so no reproducibility was needed.
Randomization	Our study is not based on field experiments, so no randomization was needed.
Blinding	Our study is not based on field experiments, so no blinding was needed.

Did the study involve field work? ☐ Yes ☒ No

Reporting for specific materials, systems and methods

We require information from authors about some types of materials, experimental systems and methods used in many studies. Here, indicate whether each material, system or method listed is relevant to your study. If you are not sure if a list item applies to your research, read the appropriate section before selecting a response.

Materials & experimental systems

n/a	Involved in the study	
☒	☐	Antibodies
☒	☐	Eukaryotic cell lines
☒	☐	Palaeontology
☒	☐	Animals and other organisms
☒	☐	Human research participants
☒	☐	Clinical data

Methods

n/a	Involved in the study	
☒	☐	ChIP-seq
☒	☐	Flow cytometry
☒	☐	MRI-based neuroimaging
